# Rotational thromboelastometry alongside conventional coagulation testing in patients with Crimean–Congo haemorrhagic fever: an observational cohort study

**DOI:** 10.1016/S1473-3099(19)30112-4

**Published:** 2019-08

**Authors:** Tom E Fletcher, Hakan Leblebicioglu, Ilkay Bozkurt, Mustafa Sunbul, Heval Bilek, Zahide Asik, Sener Barut, Ferdi Gunes, Umit Gemici, Roger Hewson, Duncan Wilson, Matt K O'Shea, Tom Woolley, Brian Faragher, Kiran Parmar, David G Lalloo, Nick J Beeching, Beverley J Hunt

**Affiliations:** a Department of Clinical Sciences, Liverpool School of Tropical Medicine, Liverpool, UK; b Tropical and Infectious Disease Unit, Royal Liverpool University Hospital, Liverpool, UK; c Ondokuz Mayis University, Samsun, Turkey; d Tokat State Hospital, Tokat, Turkey; e Gaziosmanpasa University, Tokat, Turkey; f Public Health England, Porton, UK; g Royal Centre for Defence Medicine, Birmingham, UK; h Haemostasis Research Unit, Guy's and St Thomas' Foundation Trust, London, UK

## Abstract

**Background:**

Data describing the coagulopathy of Crimean–Congo haemorrhagic fever are scarce. We did rotational thromboelastometry (ROTEM) and conventional coagulation testing in patients with Crimean–Congo haemorrhagic fever to increase our understanding of the coagulopathy of this infectious disease.

**Methods:**

We did a prospective observational cohort study of adults aged 18 years and older and admitted to hospitals with PCR-confirmed Crimean–Congo haemorrhagic fever in Samsun and Tokat, Turkey. Demographic, clinical, and laboratory data were collected and blood samples for ROTEM analysis and coagulation testing were drawn at admission and during hospital admission and convalescence (up to 30 days after onset of illness). For the ROTEM analysis we recorded the following extrinsically activated ROTEM (EXTEM S) variables, with normal ranges indicated: clotting time (38–79 s), clot formation time (34–159 s), amplitude at 10 min after clotting time (43–65 mm), maximum clot firmness (50–72 mm), and maximum lysis (>15% at 1 h). The following fibrin-specific ROTEM (FIBTEM S) variables were also recorded: amplitude at 10 min after clotting time (normal range 7–23 mm) and maximum clot firmness (9–25 mm). Disease severity was assessed by Swanepoel criteria, severity grading score (SGS), and the severity scoring index (SSI), with mild disease defined as meeting no Swanepoel criteria, graded mild by SSI, and graded low risk by SGS.

**Findings:**

Between May 27, 2015, and Aug 2, 2015, 65 patients with confirmed Crimean–Congo haemorrhagic fever were recruited and had blood taken at 110 time points. Most were male (40 [62%] of 65) with mild disease (49 [75%] of 65). Haemorrhage occurred in 13 (20%; 95% CI 11·1–31·8) of 65 patients and 23 (35%) of 65 received blood products (15 received fresh frozen plasma and eight received red blood cell concentrates), and 21 patients received platelet transfusions. At admission, the following EXTEM S variables differed significantly between mild cases and moderate to severe cases: median clotting time 56 s (range 42–81; IQR 48–64) versus 69 s (range 48–164; IQR 54–75; p=0·01); mean amplitude at 10 min after clotting time 45·1 mm (SD 7·0) versus 33·9 mm (SD 8·6; p<0·0001); median clot formation time 147 s (range 72–255; IQR 101–171) versus 197 s (range 98–418; IQR 156–296; p=0·006); and maximum clot firmness 54·4 mm (SD 7·2) versus 45·1 mm (SD 12·5; p=0·003). The EXTEM S variables were compared at different time points; maximum clot firmness (p=0·024) and amplitude at 10 min after clotting time (p=0·090) were lowest on days 4–6 of illness. We found no significant differences in FIBTEM variables between mild and moderate to severe cases (median amplitude at 10 min, 13 mm [range 8–20; IQR 11–15] *vs* 12 mm [range 6–25; IQR 10–15; p=0·68]; and median maximum clot firmness, 15 mm [range 9–60; IQR 13–21] *vs* 17 mm [range 7–39; IQR 13–23; p=0·21]); and no hyperfibrinolysis (maximum lysis >15%).

**Interpretation:**

Coagulopathy of Crimean–Congo haemorrhagic fever is related to defects in clot development and stabilisation that are more marked in severe disease than in mild disease. The combination of normal and slightly deranged coagulation screens and FIBTEM results with the absence of hyperfibrinolysis suggests that the coagulopathy of Crimean–Congo haemorrhagic fever relates to platelet dysfunction.

**Funding:**

Wellcome Trust, UK Ministry of Defence, and National Institute for Health Research Health Protection Research Unit.

## Introduction

Crimean–Congo haemorrhagic fever is a potentially fatal acute viral disease that is widespread across southern Africa and eastern Europe, Russia, and the Middle East,^1^ and is seen occasionally in travellers returning from endemic areas.^2^ Crimean–Congo haemorrhagic fever virus is transmitted to humans through tick bites or direct exposure to blood and tissues of infected animals and humans. The disease has been associated with case fatality rates of 10–40%^3^ and has been designated a priority pathogen for research and development by WHO.^4^

**Research in context**

**Evidence before this study**

We searched Medline and PubMed for studies published between May 1, 1976, and July 1, 2018, reporting clotting abnormalities in humans. We used the following keywords: “CCHF”, “Crimean-Congo Hemorrhagic Fever”, “Ebola”, “ yellow fever”, “viral haemorrhagic fever”, “dengue”, “thromboelastography”, and “thromboelastometry”, and found three peer-reviewed studies: one reporting the use of thromboelastography data for two cases of Ebola virus disease; and two cross-sectional studies of rotational thromboelastometry (ROTEM) in dengue virus infection. Before our study, Crimean–Congo haemorrhagic fever was known to be associated with substantial haemorrhage, and haemorrhagic manifestations and abnormal haematological variables have been shown to be associated with a poor clinical outcome. The evolution and pathogenesis of the coagulopathy of viral haemorrhagic fevers are poorly understood and there are few data about the coagulopathy of Crimean–Congo haemorrhagic fever.

**Added value of this study**

This study is, to the best of our knowledge, the first use of a global haemostasis test in Crimean–Congo haemorrhagic fever and the largest and most complete evaluation of ROTEM in any viral haemorrhagic fever. We show that reversible coagulopathy in Crimean–Congo haemorrhagic fever is common, haemorrhage frequently occurs in severe disease, and that traditional laboratory tests (prothrombin time and activated partial thromboplastin time) do not show substantial changes in individuals with Crimean–Congo haemorrhagic fever. These findings suggest that these tests are not detecting the cause of the coagulopathy and that bleeding is likely to be due to a defect in platelets or fibrinolysis. ROTEM shows no evidence that hyperfibrinolysis accounts for bleeding and excludes disseminated intravascular coagulation as the predominant cause of the bleeding. The defect appears to lie in the contribution of platelets to clot firmness. This study provides, to our knowledge, the first evidence that the coagulopathy in Crimean–Congo haemorrhagic fever relates to platelet dysfunction.

**Implications of all available evidence**

With no proven therapeutics available to treat Crimean–Congo haemorrhagic fever, management of coagulopathy and the associated haemorrhage is a key facet of supportive care. Global haemostasis tests such as ROTEM might improve early detection and rationalised transfusion. Future studies should investigate the platelet defect in more detail and investigate the implementation of ROTEM-guided blood product replacement.

The evolution and pathogenesis of the coagulopathy of viral haemorrhagic fevers are poorly understood, particularly for Crimean–Congo haemorrhagic fever. Concentrations of protein S and protein C, activated protein C resistance, and D-dimer were not associated with mortality in one study of 83 patients with Crimean–Congo haemorrhagic fever.^5^ Thrombocytopenia and platelet dysfunction have been shown to occur in Lassa fever^6^, ^7^, ^8^ and in Ebola virus disease.^9^

**Figure 1:**
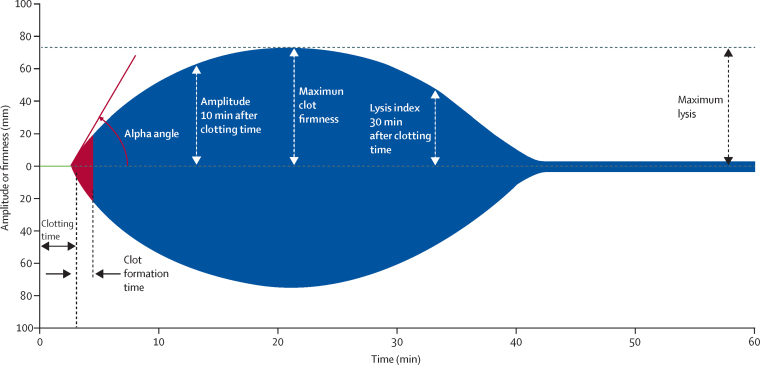
Rotational thromboelastometry (ROTEM) variables and scaling Used with permission from Instrumentation Laboratory (Bedford, MA, USA).

Traditional screening coagulation tests such as activated partial thromboplastin time provide information about coagulation factor deficiencies, but not the full haemostatic capacity of clot formation, thrombin generation, platelet number and function, or fibrinolytic activation. These tests also have a laboratory turnaround time that can delay delivery of therapeutic interventions. Rotational thromboelastometry (ROTEM) is a test with a quick turnaround time that can be done near the patient (ie, outside of laboratories) and can give a more comprehensive overview of haemostatic status. The test is derived from a technique that was first developed in 1948,^10^ and analyses three phases of coagulation (initiation, amplification, and propagation), reflecting the interactions of the cellular and plasma components of coagulation and the activity of the fibrinolytic system.^11^ A whole blood sample is placed in a cuvette and a cylindrical pin immersed, maintaining a gap bridged by the blood. The pin is rotated and, as the blood begins to clot, its movement is restricted and the changes in mechanical kinetics that result can be plotted to generate typical curves (known as TEMograms) and numerical measurements (figure 1).

ROTEM values have been used to detect and manage coagulopathy in haemorrhagic disorders, including traumatic coagulopathy, post-partum haemorrhage, intensive care, and surgery (eg, liver transplantation). ROTEM analysis of a cohort of 53 patients with dengue showed substantial impairment in thromboelastometry values.^12^ A similar technique, thromboelastography, was done on two patients with Ebola virus disease in the UK,^13^ showing evidence of a coagulopathy, followed by hypercoagulability on recovery.

We did ROTEM analysis alongside conventional coagulation testing to improve understanding of the coagulopathy of Crimean–Congo haemorrhagic fever, in the hope that these new insights would inform new approaches to improving clinical outcomes.

## Methods

### Study design

For this prospective observational cohort study, we collected demographic, clinical, and laboratory data from patients aged 18 years and older who were consecutively admitted to hospital with Crimean–Congo haemorrhagic fever in Samsun, Turkey (Ondokus Mayis University Hospital), and Tokat, Turkey (Tokat State Hospital and Gaziosmanpasa University Hospital), between May 27, 2015, and Aug 2, 2015.

The Ministry of Defence Research and Ethics Committee (UK: 571/MODREC/14) and Ondokus Mayis University Research and Ethics Committee (Turkey: OMU KAEK 2014/739) approved the research and all patients provided written informed consent.

### Procedures

Diagnosis of Crimean–Congo haemorrhagic fever was confirmed on admission by a positive Crimean–Congo haemorrhagic fever PCR result (Altona RealStar CCHFV 1.0, Hamburg, Germany) on blood samples at the Ministry of Health reference laboratory in Samsun, Turkey. Analysis of Crimean–Congo haemorrhagic fever IgM and IgG ELISA (Vectorbest, Novosibirsk, Russia) was done on serum at Public Health England, Porton, UK. Clinical features, vital signs, and laboratory test results were recorded daily on a standardised proforma with onset of disease or symptoms recorded as day 0. Blood samples for ROTEM analysis were drawn during hospital admission and during convalescence (days 14–30).

Routine haematological variables (haemoglobin, white blood cell count, platelet count, prothrombin time, and activated partial thromboplastin time) were analysed by use of automated analysers in local ISO/IEC 17025 and ISO 15189 standard accredited laboratories (Mindray, Shenzhen, China; BC-68700, Shenzhen, China; and Succeeder SF-8100, Beijing, China). The laboratory (manufacturer's) normal range for prothrombin time was 11–15 s and that for activated partial thromboplastin time was 27–45 s. Standard ROTEM analysis was done on 3·2% citrated whole blood by use of a ROTEM delta point-of-care analyser (Tem International, Munich, Germany) according to the manufacturer's instructions, less than 2 h after blood draw. Single-use extrinsically activated ROTEM (EXTEM S) and fibrin-specific ROTEM (FIBTEM S) assay reagents were used with an automated pipette for standardised volumes. Analysis was done inside a class 2a biological safety cabinet, with the operator wearing personal protective equipment.

We used EXTEM S screening, which gives information about clot formation, particularly through the extrinsic pathway, and FIBTEM S, which isolates coagulation and provides information about the contribution of fibrinogen to clot firmness. The following EXTEM S variables were recorded: clotting time (time from start of the measurement until initiation of clotting; manufacturer normal range 38–79 s), clot formation time (time from initiation of clotting until a clot firmness of 20 mm is detected; 34–159 s), amplitude at 10 min after clotting time (also known as clot firmness 10 min after clot initiation; 43–65 mm), maximum clot firmness (clot firmness and overall clot stability; 50–72 mm), and maximum lysis (>15% at 1 h). The following FIBTEM S variables were recorded: amplitude at 10 min after clotting time (manufacturer normal range 7–23 mm); and maximum clot firmness (9–25 mm). PLTEM was calculated by subtracting maximum clot firmness for FIBTEM from maximum clot firmness for EXTEM, as PLTEM might correlate better with platelet count and function than EXTEM.^14^

Severity of Crimean–Congo haemorrhagic fever was assessed in all patients by use of Swanepoel criteria,^15^ the severity grading score (SGS),^16^ and severity scoring index (SSI),^17^ for the first 5 days of illness (appendix). Patients presenting more than 5 days after onset of illness were given an SSI score at admission. They were categorised as mild if they met none of the Swanepoel criteria and were graded as mild by SSI and low risk by SGS. The moderate-to-severe group comprised all patients graded as moderate or severe on the SSI, intermediate or high risk on the SGS, or with any of the Swanepoel criteria (appendix). Missing laboratory variables were scored as zero, but severity groups and results were confirmed by singular imputation of maximal scores for patients with missing data. Patients were given blood products according to clinician assessment and blood product use was recorded daily.

### Statistical analysis

Descriptive results are reported as frequencies (proportions) for categorical variables and means (SD or 95% CI) or medians (ranges; IQR) for continuous variables. Categorical variables were compared between subgroups with the Fisher exact test. Continuous variables measured at admission were compared by use of Student's *t*-tests or the Mann-Whitney U-test or Kruskal-Wallis test as appropriate. Continuous variables measured longitudinally were compared by use of linear regression models with robust standard errors and adjustment for clustering of measures within patients. No imputation for missing data was made because of small sample sizes. Hypothesis tests were two-tailed (p<0·05) and analyses were done with SPSS, version 24, and Stata, version 14.

### Role of the funding source

The funder of the study had no role in study design, data collection, data analysis, data interpretation, or writing of the report. The corresponding author had full access to all the data in the study and had final responsibility for the decision to submit for publication.

## Results

Between May 27, 2015, and Aug 2, 2015, 65 patients with confirmed Crimean–Congo haemorrhagic fever were recruited, with 49 (75%) graded as mild severity and two (3·1%, 95% CI 0·4–10·7) of 65 patients dying. Imputation of missing data at maximal scores changed the severity grading in one patient, with no significant changes to the results. In addition to positive PCR results, Crimean–Congo haemorrhagic fever IgM and IgG ELISA was positive in 64 (98%) of 65 patients (negative IgM and IgG in one patient who died). Demographic information is provided in table 1. 212 ROTEM analyses (EXTEM S, n=107; FIBTEM S, n=105) were done on 110 blood samples grouped by day of illness: days 0–3 (n=27), days 4–6 (n=46), days 7–10 (n=25), and convalescence (n=12, median 21 days [IQR 17–30] after onset of illness). In 45 (69%) of 65 patients, baseline ROTEM analysis was done within 48 h of admission (31 [69%] of these 45 patients were graded as having mild disease). Of the remaining 20 (31%) patients, 14 (70%) had ROTEM analysis after 48 h during acute admission and six only had ROTEM analysis at follow-up during convalescence.

**Table 1:** Demographics, clinical features, and treatment characteristics of patients with Crimean–Congo haemorrhagic fever by use of ROTEM analysis

		**All cases (n=65)**	**Mild cases (n=49)**	**Moderate to severe cases (n=16)**	**p value**
Sex					
	Male	40 (62%)	31 (63%)	9 (56%)	..
	Female	25 (38%)	18 (37%)	7 (44%)	0·77
Mean age, years		53 (15·6)	53·9 (16·6)	50·3 (11·8)	0·43
Tick bite					
	Yes	45 (69%)	36 (73%)	9 (56%)	..
	No	20 (31%)	13 (27%)	7 (44%)	0·22
Mode of admission					
	Direct	56 (86%)	44 (90%)	12 (75%)	..
	Hospital transfer	9 (14%)	5 (10%)	4 (25%)	0·21
Median time from symptom onset to admission, days		2 (0–7; 2–4)	3 (1–8; 2–4)	4 (1–8; 3–6)	0·07
Median length of admission, days		8 (2–16; 6–10)	8 (3–16; 7–10)	8 (2–11; 6–9)	0·45
Fatal outcome					
	Yes	2 (3%)	0 (0%)	2 (12%)	..
	No	63 (97%)	49 (100%)	14 (88%)	0·06
Haemorrhage during admission					
	Yes	13 (20%)	7 (14%)	6 (37%)	..
	No	52 (80%)	42 (86%)	10 (63%)	0·07
Blood product replacement					
	Platelets	21 (32%)	13 (27%)	9 (56%)	0·03
	Fresh frozen plasma	15 (23%)	7 (14%)	9 (56%)	0·002
	Red blood cells	8 (12%)	3 (6%)	5 (31%)	0·02
Ribavirin treatment					
	Yes	35 (54%)	23 (47%)	12 (75%)	..
	No	30 (46%)	26 (53%)	4 (25%)	0·08

Data are n (%), mean (SD), or median (range; IQR). ROTEM=rotational thromboelastometry.

Haemorrhage occurred in 13 (20%, 95% CI 11·1–31·8) of 65 patients, who had a total of 25 days of haemorrhage during 424 days of study observation. The sites of bleeding were oral (in seven patients), nasal (in seven), sputum (in three), vomit (in two), stool (in two), intravenous puncture sites (in two), vaginal (in one), urine (in one), and skin (in one). 35 (54%) of 65 patients developed platelet counts lower than 50 × 10^9^ cells per L during admission, with 11 (17%) of 65 having a platelet count lower than 20 × 10^9^ cells per L.

Fresh frozen plasma was administered to 15 (23%) of 65 patients, red blood cell concentrates to eight (12%), and platelet transfusion to 21 (32%). 11 (85%) of 13 patients who developed haemorrhage had received blood component therapy before the onset of bleeding. At the time of bleeding (25 patient-admission days—ie, total days of bleeding during admission for the cohort), median platelet count was 44 × 10^9^ cells per L (range 4 × 10^9^ to 152 × 10^9^; IQR 17 × 10^9^ to 53 × 10^9^), median prothrombin time (only available for 24 of the 25 patient-admission days) was 13 s (range 11–38, IQR 11–14; within normal limits for 23 of 24 patients), and median activated partial thromboplastin time (only available for 24 of the 25 patient-admission days) was 48 s (range 22–85, IQR 36–56, within normal limits for ten of 24 patients). At the time of bleeding, vital signs were within normal limits for 17 (68%) of 25 patients, with only one patient having a quick sepsis-related organ failure assessment (qSOFA) score of 2 or greater and a national early warning score (NEWS) greater than 3. Ribavirin treatment was given to 35 (54%) of 65 patients at a standard dose: 30 mg/kg loading dose, then 15 mg/kg every 6 h for 4 days, and then 7·5 mg/kg every 8 h for 6 days.^18^

**Figure 2:**
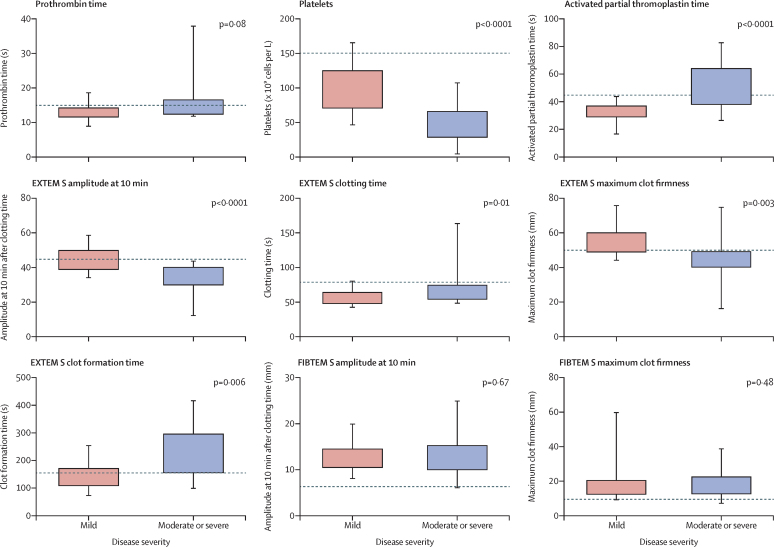
Haematological, coagulation, and rotational thromboelastometry (ROTEM) findings at admission in 45 patients with Crimean–Congo haemorrhagic fever disease, stratified by severity Boxes represent median, 25th, and 75th centiles. Whiskers represent maximum and minimum values. Dotted lines represent limits of normal ranges. EXTEM S=extrinsically activated ROTEM. FIBTEM S=fibrin-specific ROTEM.

Haematological variables on admission (n=45) are shown in table 2. Patients with moderate to severe disease had significantly lower platelet counts and more prolonged activated partial thromboplastin time than did those with mild disease. Haemoglobin, white blood cell count, and prothrombin time did not differ significantly at admission by disease severity. ROTEM analysis at admission (n=45) showed significant differences in all EXTEM S variables by disease severity, with clotting time, amplitude at 10 min after clotting time, clot formation time, and maximum clot firmness all having more abnormal values in the moderate to severe group than in the mild group (table 2; figure 2). There was no evidence of hyperfibrinolysis (>15%) at 1 h in any of the patients. Maximal clot firmness, clot formation time, PLTEM, and amplitude at 10 min after clotting time were significantly correlated with platelet count (p<0·0001; appendix). Prothrombin time correlated with clotting time (p<0·0001; appendix). For a disease thought to be associated with disseminated intravascular coagulation, amplitude at 10 min after clotting time and maximum clot firmness recorded by FIBTEM S did not differ significantly between the disease severity groups (table 2).

**Table 2:** Haematology and ROTEM data at admission (<48 h) by severity of Crimean–Congo haemorrhagic fever

		**All patients (n=45)**	**Patients with mild disease (n=31)**	**Patients with moderate to severe disease (n=14)**	**p value**
**Haematology variables**					
Platelets, × 10^9^ cells per L (range; IQR)		73 (4–162; 55–113)	90 (46–166; 71–125)	46 (4–108; 29–66)	<0·0001
White blood cells, × 10^9^ cells per L (range; IQR)		2·4 (0·74–28·3; 1·53–3·35)	2·35 (0·74–5·31; 1·48–3·11)	2·45 (0·85–28·3; 1·52–3·70)	0·74
Prothrombin time, s*		14·0 (4·4)	13·1 (2·1)	15·8 (7·0)	0·08
Activated partial thromboplastin time, s*		38·3 (13·8)	32·8 (6·7)	49·9 (17·8)	<0·0001
Haemoglobin, g/dL		13·5 (2·1)	13·8 (1·8)	12·7 (2·6)	0·10
**ROTEM (EXTEM S) variables**					
Clotting time, s					
	Median (range; IQR)	59 (42–164; 49–69)	56 (42–81; 48–64)	69 (48–164; 54–75)	0·01
	38–79 s	42	30	12	..
	>79 s	3	1	2	..
Amplitude at 10 min, mm					
	Mean (SD)	41·6 (9·1)	45·1 (7·0)	33·9 (8·6)	<0·0001
	43–65 mm	19	17	2	..
	<43 mm	26	14	12	..
Clot formation time, s					
	Median (range; IQR)	165 (72–418; 130–201)	147 (72–255; 109–171)	197 (98–418; 156–296)	0·006
	34–159 s (normal range)	20	16	4	..
	160–220 s (usually unimpaired haemostasis with reduced n reserve)	17	13	4	..
	221–300 s (bleeding risk)	6	2	4	..
	301–400 s (high bleeding risk)	0	0	0	..
	>400 s (usually no effective haemostasis)	2	0	2	..
Maximum clot firmness, mm					
	Mean (SD)	51·5 (10·1)	54·4 (7·2)	45·1 (12·5)	0·003
	≥50 mm (normal range)	24	21	3	..
	46–49 mm (usually unimpaired haemostasis with reduced n reserve)	12	7	5	..
	40–45 mm (bleeding risk)	6	3	3	..
	30–39 mm (high bleeding risk)	2	0	2	..
	<30 mm (usually no effective haemostasis)	1	0	1	..
Lysis index at 60 min >15%		0	0	0	NA
**ROTEM (FIBTEM S) variables**					
Amplitude at 10 min, mm					
	Median (range; IQR)	13 (6–25; 10–15)	13 (8–20; 11–15)	12 (6–25; 10–15)	0·68
	7–23 mm	42	29	13	..
	<7 mm	1	0	1	..
Maximum clot firmness, mm					
	Median (range; IQR)	15 (7–60; 13–21)	15 (9–60; 13–21)	17 (7–39; 13–23)	0·21
	9–25 mm	41	29	12	..
	<9 mm	2	0	2	..

Data are n, mean (SD), or median (range; IQR). NA=not available. ROTEM=rotational thromboelastometry. EXTEM S=extrinsically activated ROTEM. FIBTEM S=fibrin-specific ROTEM.

* 28 patients in mild group and 13 in moderate to severe group.

During hospital admission, among the patients with EXTEM S clot formation time measurements that showed increased bleeding risk (>221 s), 17 (71%) of 24 patients had platelet counts greater than 20 × 10^9^ cells per L and five (21%) of 24 had counts greater than 50 × 10^9^ cells per L. Of the patients with EXTEM S maximum clot firmness values showing bleeding risk (<46 mm), 18 (75%) of 24 patients had platelet counts greater than 20 × 10^9^ cells per L and five (21%) of 24 had platelet counts greater 50 × 10^9^ cells per L, again suggesting that the platelet count was not the determining factor of bleeding risk (appendix).

Clotting time did not differ significantly by day of illness (p=0·68; appendix). Results of the EXTEM S analysis of amplitude at 10 min after clotting time and maximum clot firmness differed by day of illness, with lowest values occurring on day 4 (p=0·090) and day 6 of illness (p=0·024; appendix). The EXTEM S lysis index at 60 min did not differ significantly by day of illness, with no abnormal results. FIBTEM S amplitude at 10 min after clotting time showed significant differences by day of illness in acute samples, with days 0–3 and 4–6 having the lowest values (p=0·003), but only one of 93 fibrinogen values was lower than normal (<7 s). FIBTEM S maximum clot firmness was also lowest on days 0–3 and 4–6 (p=0·082 for the three timepoints evaluated together by linear regression models). All EXTEM S and FIBTEM S variables in convalescence (n=12) were within normal ranges. EXTEM S variables of clotting time (p=0·013), amplitude at 10 min after clotting time (p<0·001), clot formation time (p<0·001), and maximum clot firmness (p<0·001) were significantly worse in acute versus convalescent samples, but FIBTEM S did not differ significantly between acute and convalescent samples.

**Figure 3:**
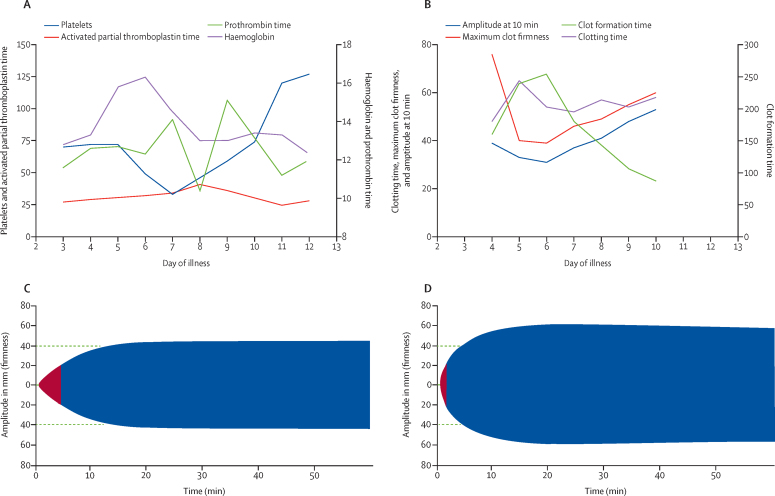
Illustrative case with serial ROTEM analyses Case history: male patient aged 30 years, admitted 3 days after a tick bite with a 1-day history of fever, lethargy, headache, and anorexia. The patient developed gingival bleeding on days 7 and 8 of illness and was discharged on day 11 of illness. The patient did not receive any blood products during admission. (A) Haematological and coagulation variables. (B) ROTEM EXTEM S variables by day of illness. (C) EXTEM S TEMogram at day 6 of illness. (D) ROTEM EXTEM S TEMogram on day 10 of illness. EXTEM=extrinsically activated ROTEM. ROTEM=rotational thromboelastometry.

Details of a typical case of Crimean–Congo haemorrhagic fever, with ROTEM data, standard haematological and coagulation variables, and TEMograms, is shown in figure 3. Two patients died. The first was a man aged 69 years who had no episodes of bleeding, normal ROTEM variables, and died from a hospital-acquired infection (*Staphylococcus aureus* septicaemia). The second was a woman aged 52 years who presented on day 4 of illness and then rapidly developed septic shock with minor bleeding from the oral mucosa, nose, and intravenous access sites. Bloods results at admission for the second patient were as follows: activated partial thromboplastin time 120 s, prothrombin time 40 s, and platelets 28 × 10^9^ cells per L. ROTEM values at admission were highly abnormal: EXTEM S clotting time 164 s, amplitude at 10 min after clotting time 33 mm, clot formation time 240 s, maximum clot firmness 46 mm, lysis index at 60 min less than 15% (no evidence of hyperfibrinolysis), FIBTEM amplitude at 10 min after clotting time 7 mm, and maximum clot firmness 7 mm. The second patient developed multiorgan failure, dying within 36 h of admission, despite ribavirin treatment. The patient received two units of platelets and six units of fresh frozen plasma on each day of admission.

## Discussion

Crimean–Congo haemorrhagic fever is a major emerging infectious disease threat and, although ribavirin is frequently used, the mainstay of case management is supportive care. Haemorrhage often occurs in severe disease and traditional laboratory tests (prothrombin time and activated partial thromboplastin time) do not show substantial abnormalities. This finding suggests that the coagulopathy seen in patients with Crimean–Congo haemorrhagic fever is not due to disseminated intravascular coagulation but rather due to defects in platelet number or function, or fibrinolysis. This study is, to the best of our knowledge, the first to investigate the use of a global haemostasis test, ROTEM, alongside conventional testing in Crimean–Congo haemorrhagic fever.

Results of traditional coagulation tests were within the normal range of values and yet many patients had bleeding. On admission, most patients (42 [93%] of 45) had normal EXTEM S clotting times, showing that initiation of clotting, thrombin formation, and the start of clot polymerisation were not significantly abnormal in most patients at this point. By contrast, EXTEM S clot formation times were abnormal in most patients at admission. Clot formation time represents the time from initiation of clotting until a clot firmness of 20 mm is detected and elevated results indicate problems with fibrin polymerisation and stabilisation of the clot with factor XIII and platelets. In the face of normal or slightly deranged activated partial thromboplastin time and prothrombin time, this result points to a platelet or factor XIII defect.

In keeping with the results of clot formation time, maximum clot firmness was abnormal in about half of patients on admission and was highly deranged in a fifth. Abnormal values for maximum clot firmness indicate reduced stabilisation of the clot by the polymerised fibrin, platelets, and factor XIII. Significant activation of fibrinolysis can be discounted as no significant reduction was observed in maximum clot firmness or clot lysis in any patient. In FIBTEM S, coagulation is activated as in EXTEM S but the addition of cytochalasin D, an antiplatelet agent, blocks the effects of platelets. The resulting clot formation therefore depends only on fibrin formation and fibrin polymerisation, which mainly relates to fibrinogen concentrations. Our results show that, at admission, most patients had a normal FIBTEM S amplitude at 10 min after clotting time and maximum clot firmness, indicating normal fibrinogen concentrations. Disseminated intravascular coagulation is characterised by consumption of coagulation factors, with the formation of small thrombi throughout the vasculature leading to end organ failure. A key finding in disseminated intravascular coagulation is the presence of hypofibrinogenaemia and activation of fibrinolysis. The absence of significantly low fibrinogen concentrations and of activation of fibrinolysis are therefore out of keeping with a diagnosis of disseminated intravascular coagulation.

We detected a reversible coagulopathy in patients who survived Crimean–Congo haemorrhagic fever. Combined data from acute samples showed significant differences in all EXTEM S variables compared with those in convalescent samples taken after discharge in a subset of survivors, which showed ROTEM results within normal ranges. When acute samples were stratified by day of illness, days 4–6 had the most abnormal values, indicating that this is the major coagulopathic period in Crimean–Congo haemorrhagic fever with respect to a patient's ability to generate stable clots. Although we observed no significant difference in median clotting time, the highest percentage of abnormal clotting time results was also found in days 4–6. This finding is consistent with previous reports and consensus that patients with severe Crimean–Congo haemorrhagic fever begin to haemorrhage during days 3–6.^19^

We plan to investigate factor XIII further but consider it unlikely to be the cause of the bleeding defect because of the clinical pattern of bleeding from mucous membranes, which is in keeping with a platelet defect. This defect was not simply due to thrombocytopenia, as patients with platelet counts higher than 50 × 10^9^ per L did not have spontaneous bleeding.

The two viral haemorrhagic fevers investigated for platelet dysfunction (Lassa fever and Argentinian haemorrhagic fever) have shown a clear platelet function defect on platelet aggregometry.^8^, ^20^ It is logical to consider that all viral haemorrhagic fevers will have similar pathogenic pathways and produce similar patterns of bleeding. The platelet defect could be due to a change in von Willebrand factor, possibly through the loss of large molecular weight forms. However, this possibility is unlikely, as a change in von Willebrand factor is usually either due to high shear stresses or an antibody against von Willebrand factor, and has never been associated with a viral disease. We believe our findings exclude disseminated intravascular coagulation as the cause of the bleeding defect in patients presenting with Crimean–Congo haemorrhagic fever. Disseminated intravascular coagulation is characterised by a consumptive coagulopathy associated with low platelet counts (which we found in our analysis), as well as low FIBTEM values, prolonged prothrombin time, and activated partial thromboplastin time, with prolongation of clotting time, all reflecting low coagulation factors^21^: these were not seen in this study. However, as shown by the second patient who died in our study, disseminated intravascular coagulation can occur in fatal cases of Crimean–Congo haemorrhagic fever associated with preterminal multiorgan failure.

A limitation of our study is that fewer patients with severe or fatal disease were recruited than expected, but we were able to show that the coagulopathy occurred in those with mild disease. The clinical and laboratory data we report are consistent with those from larger cohorts in Turkey in which prothrombin time prolongation occurred in 14 (3%) of 404 patients and an activated partial thromboplastin time longer than 70 s was seen in 15 (4%) of 404 patients.^16^ Crimean–Congo haemorrhagic fever severity scores were developed to predict mortality and have been validated in health-care settings in Turkey^16^, ^17^ where there is access to good supportive care and blood transfusion. In this study, some patients had data missing at admission for severity score calculation, which potentially risked underscoring of some patients. However, data imputation with maximum and minimum scores did not change the severity grouping of most patients or alter the significance of the results (data not shown). Prognostic scoring at baseline is challenging and existing scoring systems require laboratory variables, which are not routinely available in all settings. In routine practice, simple variables such as a platelet count lower than 50 × 10^9^ cells per L are used as the trigger for referral to tertiary centres, indicating more severe disease, increased likelihood of haemorrhage, and the possible need for blood transfusion.^3^

Given the observational nature of the study we did not do multivariate analysis or analyse data stratified by subsequent ribavirin use. Likewise, we could not analyse data on blood component therapy use in relation to ROTEM variables. The decision to use blood components was up to the treating clinicians with non-protocolised practice. ROTEM analysis was also not routinely done at specific timepoints after transfusion to allow evaluation of the effect of transfusion on haemostasis, which we plan to evaluate in future studies. We have shown that use of ROTEM in Crimean–Congo haemorrhagic fever is feasible and can provide safe and rapid point-of-care results. A limitation of ROTEM is that it does not reflect what happens in blood vessels because of its inability to reproduce the endothelium. Although ROTEM is unable to confirm platelet dysfunction or factor XIII deficiency, potential defects can be inferred from ROTEM data and clinical patterns of bleeding. Serial daily ROTEM analysis of all cases would provide more complete data, but we were limited by the practical challenges of time required for analysis, high caseloads, and safety considerations. Earlier recruitment of patients during the course of the illness would have provided more data about the progression of the coagulopathy, but was limited by delays in presentation and the time required for recruiting patients and obtaining informed consent.

Management of Crimean–Congo haemorrhagic fever is challenging because few therapeutics are available and the infection is associated with high mortality. Our ROTEM and conventional coagulation analysis has shown that the coagulopathy of Crimean–Congo haemorrhagic fever is predominantly related to clot development and stabilisation that is most marked during days 4–6 of the illness and in severe disease. In the context of normal or only slightly deranged coagulation screens and FIBTEM results, and combined with the absence of hyperfibrinolysis, this study provides the first evidence that the initial coagulopathy seen in Crimean–Congo haemorrhagic fever probably relates to platelet dysfunction. Further studies are required to confirm these findings and explore possible mechanisms for this platelet dysfunction.
