# Supplementary material for: Rotational thromboelastometry alongside conventional coagulation testing in patients with Crimean–Congo haemorrhagic fever: an observational cohort study
Source: Lancet Infect Dis. 2019 Aug;19(8):862–71. doi: 10.1016/S1473-3099(19)30112-4 (PMC7641897; doi:10.1016/S1473-3099(19)30112-4)
Supplement: Supplementary appendix [file mmc1.pdf]

THE LANCET Infectious Diseases

Supplementary webappendix

This webappendix formed part of the original submission and has been peer reviewed. We post it as supplied by the authors.

Supplement to: Fletcher TE, Leblebicioglu H, Bozkurt I, et al. Rotational thromboelastometry alongside conventional coagulation testing in patients with Crimean–Congo haemorrhagic fever: an observational cohort study. *Lancet Infect Dis* 2019; published online June 28. http://dx.doi.org/10.1016/S1473-3099(19)30112-4.

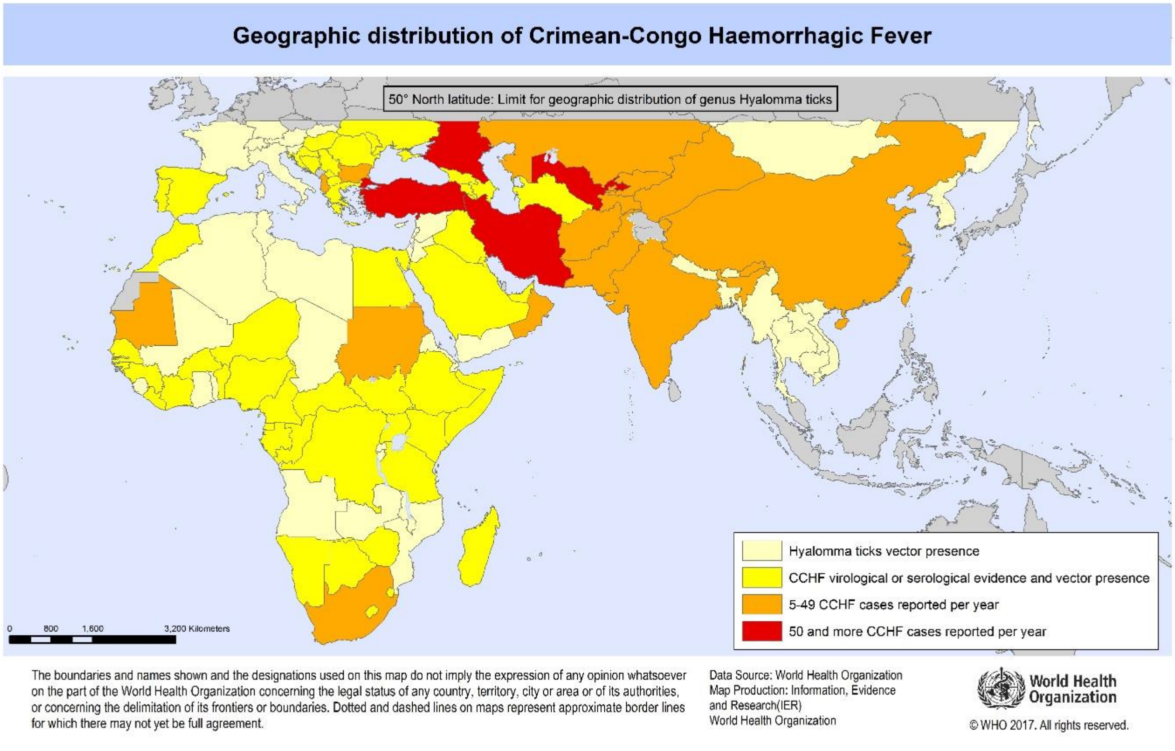

Supplementary figure 1. Geographic distribution of CCHF (reproduced with permission WHO).

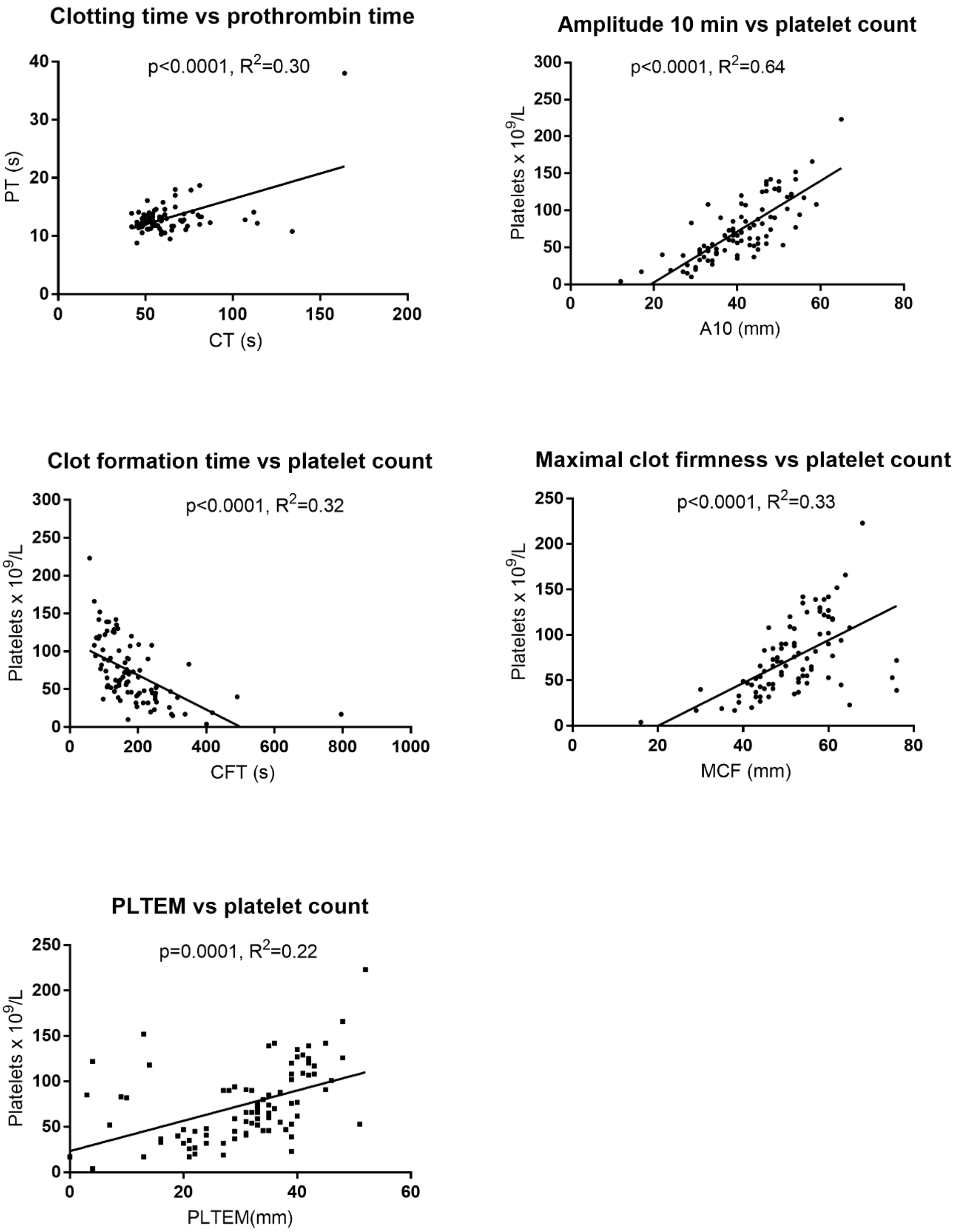

Supplementary figure 2: ROTEM and conventional coagulation/haematology correlations

**Swanepoel criteria**<br>- 1st 5 days of illness<br>- Any of following >90% predictive fatal outcome	**Severity grading system**<br>- 1st 5 days of illness<br>- 0-4 Low risk<br>- 5-8 Intermediate risk<br>- >9 High risk	**Severity scoring index**<br>- At admission<br>- 0-2 Mild<br>- 3-9 Moderate<br>- 10-13 Severe
Platelet count ≤20 X 10^9 /L	Platelet count<br>≥100,000 cells/µL = 0<br>≥50,000, <100,000 cells/µL = 1<br><50,000 cells/µL = 2	Platelet count (×10^3/mm^3)<br>>150 = 0<br>150–50 = 1<br>49–20 = 2<br><20 = 3
APTT ≥ 60 seconds	aPTT (seconds)<br><70 = 0<br>≥70 = 1	aPTT (seconds)<br>≤34 = 0<br>35–45 = 1<br>46–59 = 2<br>>60 = 3
Aspartate transaminase ≥200 U/L	Aspartate transaminase<br>< 5×ULNV = 0<br>≥ 5×ULNV = 1<br>Lactate dehydrogenase<br><3×ULNV = 0<br>≥3×ULNV= 1	Fibrinogen level, mg/dL<br>≥180 = 0<br>179–160 = 1<br>159–120 = 2<br><120 = 3
Alanine transaminase ≥150 U/L	Alanine transaminase<br><ULNV = 0<br>≥ULNV = 1	Bleeding<br>No = 0<br>Petechia = 1<br>Ecchymosis = 2<br>Bleeding = 3
White blood cells ≥10,000 cells/µL	White blood cells<br><10,000 cells /µL = 0<br>≥10,000 cells /µL = 1	Somnolence<br>No = 0<br>Yes = 1
Fibrinogen ≥110 mg/dL.	Organ failure<br>No = 0<br>Yes = 1	
	Bleeding<br>No = 0<br>Yes = 1	
	Age<br><60 = 0<br>>60 = 1	
	Prolongation of PT<br><3 s = 0<br>≥3s, <6 s = 1<br>≥6s = 2	
	Hepatomegaly<br>No = 0<br>Yes = 1	
	INR<br><1.6 = 0<br>≥1.6 = 1	

Supplementary Table 1: CCHF severity scoring systems

ROTEM parameter		Acute Day 1-3	Acute Day 4-6	Acute Day 7-10	p-value	Acute all	Convalescent Day 14-30	p-value
EXTEM S CT (n=107)	Mean (95% CI)	61.0 (55.8 : 66.2)	64.1 (56.5 : 71.6)	59.7 (52.1 : 67.3)	0.686	62.1 (57.6 : 66.6)	50.7 (47.0 : 54.3)	<0.001
38-79	n	24	38	21		83	13	
>79	n	2	6	3		11	0	
EXTEM S A10 (n=107)	Mean (95% CI)	42.5 (39.0 : 46.1)	37.9 (34.6 : 41.2)	42.6 (38.3 : 46.9)	0.090	40.4 (38.3 : 42.5)	62.9 (58.3 – 67.5)	<0.001
43-65	n	11	15	13		39	13	
<43	n	15	29	11		55	0	
EXTEM S CFT (n=107)	Mean (95% CI)	168 (137 : 200)	210 (169 : 250)	155 (124 : 187)	0.097	184 (161 : 207)	71 (55 : 88)	<0.001
34 - 159 (normal range)	n	10	18	15		43	13	
160 - 220 (usually unimpaired haemostasis with reduced reserve)	n	12	10	5		27	0	
221 - 300 (bleeding risk)	n	3	10	3		16	0	
300 - 400 (high bleeding risk)	n	0	3	1		4	0	
>400 (usually no effective haemostasis)	n	1	3	0		4	0	
EXTEM S MCF (n=102)	Mean (95% CI)	52.3 (48.7 : 55.9)	48.7 (45.2 : 52.2)	55.5 (52.2 : 58.8)	0.024	51.5 (49.4 : 53.5)	68.5 (64.8 : 72.3)	<0.001
>50 (normal range)	n	13	22	16		47	13	
46-49 (usually unimpaired haemostasis with reduced reserve)	n	9	5	2		16	0	
40-45 (bleeding risk)	n	3	9	4		16	0	
30-40 (high bleeding risk)	n	1	4	1		6	0	
<30 (usually no effective haemostasis)	n	0	2	0		4	0	
EXTEM S LI60 (n=93)	Mean (95% CI)	95.0 (93.6 : 96.4)	97.9 (96.9 : 98.8)	96.4 (94.4 : 98.4)	<0.001	96.7 (95.8 : 97.7)	94.8 (93.6 : 96.0)	0.021
<15% (normal range)	n	22	40	19		81	12	
>15%	n	0	0	0		0	0	
FIBTEM S A10 (n=105)	Mean (95% CI)	12.7 (11.6 : 13.9)	14.1 (12.6 : 15.7)	16.2 (14.6 : 17.8)	0.003	14.3 (13.3 : 15.3)	16.8 (13.5 : 20.1)	0.146
>7 (normal range 7-23)	n	24	43	24		91	13	
<7	n	1	0	0		1	0	
FIBTEM S MCF (n=105)	Mean (95% CI)	17.0 (13.1 : 20.9)	20.2 (16.9 : 23.5)	23.8 (19.8 : 27.7)	0.082	20.3 (18.0 : 22.5)	22.4 (13.4 : 31.3)	0.650
>9 (normal range 9-25)	n	24	42	24		90	13	
<9	n	1	1	0		2	0	

Supplementary Table 2. ROTEM Thromboelastometry data by grouped day of acute illness and acute vs convalescent samples

95%CI: 95% confidence interval
